# Supplementary material for: The application of virtual teams in the improvement of enterprise management capability from the perspective of knowledge transfer
Source: PLoS One. 2022 Mar 23;17(3):e0264367. doi: 10.1371/journal.pone.0264367 (PMC8942250; doi:10.1371/journal.pone.0264367)
Supplement: Attachment — Submitted filename: Response to Reviewers.docx [file pone.0264367.s003.docx]

**7.13**

PONE-D-20-35199
The Application of Virtual Teams in the Improvement of Enterprise Management Capability from the Perspective of Knowledge Transfer
PLOS ONE

Dear Dr. Zhang,

Thank you for submitting your manuscript to PLOS ONE. After careful consideration, we feel that it has merit but does not fully meet PLOS ONE’s publication criteria as it currently stands. Therefore, we invite you to submit a revised version of the manuscript that addresses the points raised during the review process.

Please submit your revised manuscript by Aug 26 2021 11:59PM. If you will need more time than this to complete your revisions, please reply to this message or contact the journal office at [plosone@plos.org](mailto:plosone@plos.org). When you're ready to submit your revision, log on to <https://www.editorialmanager.com/pone/> and select the 'Submissions Needing Revision' folder to locate your manuscript file.

Please include the following items when submitting your revised manuscript:

- A rebuttal letter that responds to each point raised by the academic editor and reviewer(s). You should upload this letter as a separate file labeled 'Response to Reviewers'.
- A marked-up copy of your manuscript that highlights changes made to the original version. You should upload this as a separate file labeled 'Revised Manuscript with Track Changes'.
- An unmarked version of your revised paper without tracked changes. You should upload this as a separate file labeled 'Manuscript'.

If you would like to make changes to your financial disclosure, please include your updated statement in your cover letter. Guidelines for resubmitting your figure files are available below the reviewer comments at the end of this letter.

If applicable, we recommend that you deposit your laboratory protocols in protocols.io to enhance the reproducibility of your results. Protocols.io assigns your protocol its own identifier (DOI) so that it can be cited independently in the future. For instructions see: <http://journals.plos.org/plosone/s/submission-guidelines#loc-laboratory-protocols>. Additionally, PLOS ONE offers an option for publishing peer-reviewed Lab Protocol articles, which describe protocols hosted on protocols.io. Read more information on sharing protocols at <https://plos.org/protocols?utm_medium=editorial-email&utm_source=authorletters&utm_campaign=protocols>.

We look forward to receiving your revised manuscript.

Kind regards,

Alessandro Margherita
Academic Editor
PLOS ONE

Journal Requirements:

When submitting your revision, we need you to address these additional requirements.

1. Please ensure that your manuscript meets PLOS ONE's style requirements, including those for file naming. The PLOS ONE style templates can be found at

<https://journals.plos.org/plosone/s/file?id=wjVg/PLOSOne_formatting_sample_main_body.pdf> and

https://journals.plos.org/plosone/s/file?id=ba62/PLOSOne_formatting_sample_title_authors_affiliations.pdf

Reply: We have ensured that this manuscript conforms to the publishing style of PLOS One.

2. PLOS requires an ORCID iD for the corresponding author in Editorial Manager on papers submitted after December 6th, 2016. Please ensure that you have an ORCID iD and that it is validated in Editorial Manager. To do this, go to ‘Update my Information’ (in the upper left-hand corner of the main menu), and click on the Fetch/Validate link next to the ORCID field. This will take you to the ORCID site and allow you to create a new iD or authenticate a pre-existing iD in Editorial Manager. Please see the following video for instructions on linking an ORCID iD to your Editorial Manager account:<https://www.youtube.com/watch?v=_xcclfuvtxQ>

Reply: We have ensured that the ORCID ID is correct and verified.

3. Please ensure that you include a title page within your main document. You should list all authors and all affiliations as per our author instructions and clearly indicate the corresponding author.

Reply: We have ensured that the title of the article and the affiliation of the author are correct.

4. Please amend either the title on the online submission form (via Edit Submission) or the title in the manuscript so that they are identical.

Reply: We have ensured that the title of the submitted manuscript is consistent with the title in the online form.

[Note: HTML markup is below. Please do not edit.]

Reviewers' comments:

Reviewer's Responses to Questions

**Comments to the Author**

1. Is the manuscript technically sound, and do the data support the conclusions?

The manuscript must describe a technically sound piece of scientific research with data that supports the conclusions. Experiments must have been conducted rigorously, with appropriate controls, replication, and sample sizes. The conclusions must be drawn appropriately based on the data presented.

Reviewer #1: No

Reviewer #2: Partly

Reply: Thanks for the careful reading. In the empirical research part, we carried out more specific research and appropriate control, and the research results support the conclusion.

2. Has the statistical analysis been performed appropriately and rigorously?

Reviewer #1: I Don't Know

Reviewer #2: Yes

Reply: Thanks for the careful reading. We conducted rigorous statistical analysis in ensuring empirical research.

3. Have the authors made all data underlying the findings in their manuscript fully available?

The [PLOS Data policy](http://www.plosone.org/static/policies.action#sharing) requires authors to make all data underlying the findings described in their manuscript fully available without restriction, with rare exception (please refer to the Data Availability Statement in the manuscript PDF file). The data should be provided as part of the manuscript or its supporting information, or deposited to a public repository. For example, in addition to summary statistics, the data points behind means, medians and variance measures should be available. If there are restrictions on publicly sharing data—e.g. participant privacy or use of data from a third party—those must be specified.

Reviewer #1: No

Reviewer #2: No

Reply: Thanks for the careful reading. Our data comes from the public data of enterprise a, and the openness and reliability of the data are described in the text.

4. Is the manuscript presented in an intelligible fashion and written in standard English?

PLOS ONE does not copyedit accepted manuscripts, so the language in submitted articles must be clear, correct, and unambiguous. Any typographical or grammatical errors should be corrected at revision, so please note any specific errors here.

Reviewer #1: No

Reviewer #2: Yes

Reply: Thanks for the careful reading. We have ensured that the English expression of the manuscript is correct.

5. Review Comments to the Author

Please use the space provided to explain your answers to the questions above. You may also include additional comments for the author, including concerns about dual publication, research ethics, or publication ethics. (Please upload your review as an attachment if it exceeds 20,000 characters)

Reviewer #1: This article covers the importance of virtual teams and the process of knowledge transfer. The article presents original ideas, for example, the representation of virtual teams as flat dynamic networks.
Reply: Thank you for your summary of this study. This study puts forward a set of schemes conducive to virtual team cooperation.
Overall, the article could be better organized. The sections of the text need to be more developed and some of the sentences are not completely clear. For example, “The term “virtual” … refers to technology that does not exist but has physical functions.”

Reply: We have improved the English expression in the paper and made clear the organization and expression of sentences.
Some ideas need clarification -- “Before the project is implemented, the project development team and the project manager need to be divided into a large business department to eliminate communication barriers.” Is this about a larger team or a division into smaller teams? Is the goal to divide or expand? What exactly is the paper suggesting as a solution?
Reply: We have clarified the statement in this sentence that members need to build a team around the same issue.
Regarding the Evaluation of R&D and innovation technology capabilities of virtual teams, the data used for the neural network is not available. The process of using a neural network is not described in detail. Data samples used to train the neutral network are missing. Six enterprises are listed in the “Evaluation of R&D and innovation technology capabilities of virtual teams”. There is no explanation of the choice of indexes used for the six enterprises. The methodology is difficult to analyze. Some common-sense indexes such as experience in the field, references from prior work or trust which could greatly influence a ranking of the enterprises are not mentioned in the evaluation. In conclusion, I think that a comparison between the case model using a neural network and the case model without the use of a neural network could help the reader understand the advantages of using neural networks for the evaluation.

Reviewer #2: The authors should:
1) define more clearly the research gap (in the extant literature) already in the introduction
2) present in a more comprehensive way (e.g. using a chart/process) the research roadmap undertaken
3) distinguish better what, in section 2, represents theory background and which one are arguments/speculations deriving from the same
4) provide more background on the analysed case and justify selection of the same
5) build a section with clear theoretical advancements and practitioner implications of the study

Reply: Thanks for the suggestion. (1) We have explained the existing research gaps in the introduction; (2) We have explained the specific process of the research in the introduction, which is not suitable for flow chart display; (3) We have explained in detail the virtual team cooperation theory in the second part; (4) We have explained more research background related to the issues discussed in this paper; (5) We have supplemented the theoretical progress and practical significance in the conclusion.

6. PLOS authors have the option to publish the peer review history of their article ([what does this mean?](https://journals.plos.org/plosone/s/editorial-and-peer-review-process#loc-peer-review-history)). If published, this will include your full peer review and any attached files.
If you choose “no”, your identity will remain anonymous but your review may still be made public.
Reply: Our paper can be published and peer reviewed.

**Do you want your identity to be public for this peer review?** For information about this choice, including consent withdrawal, please see our [Privacy Policy](https://www.plos.org/privacy-policy).

Reviewer #1: No

Reviewer #2: No

**9.28**

PONE-D-20-35199R1

The Application of Virtual Teams in the Improvement of Enterprise Management Capability from the Perspective of Knowledge Transfer

PLOS ONE

Dear Dr. Zhang,

Thank you for submitting your manuscript to PLOS ONE. After careful consideration, we feel that it has merit but does not fully meet PLOS ONE’s publication criteria as it currently stands. Therefore, we invite you to submit a revised version of the manuscript that addresses the points raised during the review process.

It is not really clear at what extent the authors have addressed all the comments provided by the two reviewers. If we look at the amendments made in the revised text (i.e. the red coloured parts), it seems that the revisions are quite limited for a double "major revision". Also the explanations provided by the authors are not enough detailed to let understand where and how they brought changes to the original version. The authors are thus required to go once again through all the reviewers' comments and to provide a more extended and convincing discussion of the revision work undertaken.

Please submit your revised manuscript by Nov 11 2021 11:59PM. If you will need more time than this to complete your revisions, please reply to this message or contact the journal office at [plosone@plos.org](mailto:plosone@plos.org). When you're ready to submit your revision, log on to <https://www.editorialmanager.com/pone/> and select the 'Submissions Needing Revision' folder to locate your manuscript file.

Please include the following items when submitting your revised manuscript:

- A rebuttal letter that responds to each point raised by the academic editor and reviewer(s). You should upload this letter as a separate file labeled 'Response to Reviewers'.
- A marked-up copy of your manuscript that highlights changes made to the original version. You should upload this as a separate file labeled 'Revised Manuscript with Track Changes'.
- An unmarked version of your revised paper without tracked changes. You should upload this as a separate file labeled 'Manuscript'.

If you would like to make changes to your financial disclosure, please include your updated statement in your cover letter. Guidelines for resubmitting your figure files are available below the reviewer comments at the end of this letter.

If applicable, we recommend that you deposit your laboratory protocols in protocols.io to enhance the reproducibility of your results. Protocols.io assigns your protocol its own identifier (DOI) so that it can be cited independently in the future. For instructions see: <https://journals.plos.org/plosone/s/submission-guidelines#loc-laboratory-protocols>. Additionally, PLOS ONE offers an option for publishing peer-reviewed Lab Protocol articles, which describe protocols hosted on protocols.io. Read more information on sharing protocols at <https://plos.org/protocols?utm_medium=editorial-email&utm_source=authorletters&utm_campaign=protocols>.

We look forward to receiving your revised manuscript.

Kind regards,

Alessandro Margherita
Academic Editor
PLOS ONE


[Note: HTML markup is below. Please do not edit.]

Reviewers' comments:


[NOTE: If reviewer comments were submitted as an attachment file, they will be attached to this email and accessible via the submission site. Please log into your account, locate the manuscript record, and check for the action link "View Attachments". If this link does not appear, there are no attachment files.]

While revising your submission, please upload your figure files to the Preflight Analysis and Conversion Engine (PACE) digital diagnostic tool, <https://pacev2.apexcovantage.com/>. PACE helps ensure that figures meet PLOS requirements. To use PACE, you must first register as a user. Registration is free. Then, login and navigate to the UPLOAD tab, where you will find detailed instructions on how to use the tool. If you encounter any issues or have any questions when using PACE, please email PLOS at [figures@plos.org](mailto:figures@plos.org). Please note that Supporting Information files do not need this step.

Reply: We have comprehensively checked the article and confirmed the information, and the submitted manuscript meets the requirements of PLoS One.
